# Studying Dynamic Features in Myocardial Infarction Progression by Integrating miRNA-Transcription Factor Co-Regulatory Networks and Time-Series RNA Expression Data from Peripheral Blood Mononuclear Cells

**DOI:** 10.1371/journal.pone.0158638

**Published:** 2016-07-01

**Authors:** Hongbo Shi, Guangde Zhang, Jing Wang, Zhenzhen Wang, Xiaoxia Liu, Liang Cheng, Weimin Li

**Affiliations:** 1 College of Bioinformatics Science and Technology, Harbin Medical University, Harbin, Heilongjiang, 150081, PR China; 2 Department of Cardiology, The Fourth Affiliated Hospital of Harbin Medical University, Harbin, Heilongjiang, 150001, PR China; 3 Department of Cardiology, The First Affiliated Hospital of Harbin Medical University, Harbin, Heilongjiang, 150001, PR China; West China Second Hospital, Sichuan University, CHINA

## Abstract

Myocardial infarction (MI) is a serious heart disease and a leading cause of mortality and morbidity worldwide. Although some molecules (genes, miRNAs and transcription factors (TFs)) associated with MI have been studied in a specific pathological context, their dynamic characteristics in gene expressions, biological functions and regulatory interactions in MI progression have not been fully elucidated to date. In the current study, we analyzed time-series RNA expression data from peripheral blood mononuclear cells. We observed that significantly differentially expressed genes were sharply up- or down-regulated in the acute phase of MI, and then changed slowly until the chronic phase. Biological functions involved at each stage of MI were identified. Additionally, dynamic miRNA–TF co-regulatory networks were constructed based on the significantly differentially expressed genes and miRNA–TF co-regulatory motifs, and the dynamic interplay of miRNAs, TFs and target genes were investigated. Finally, a new panel of candidate diagnostic biomarkers (STAT3 and ICAM1) was identified to have discriminatory capability for patients with or without MI, especially the patients with or without recurrent events. The results of the present study not only shed new light on the understanding underlying regulatory mechanisms involved in MI progression, but also contribute to the discovery of true diagnostic biomarkers for MI.

## Introduction

Myocardial infarction (MI) is defined pathologically as myocardial cell death caused by prolonged ischemia, and is a leading cause of morbidity, mortality and cost to society [1]. The recurrence of MI greatly increases the risk of death. Clinically, different methods are used for diagnosis, including electrocardiography, blood tests, and coronary angiography.

In the past decades, the molecular mechanisms underlying MI have been widely investigated. Most of these studies focused on several genes on specific conditions. For example, glutaredoxin regulates apoptosis in cardiomyocytes via NFκB targeting Bcl-2 and Bcl-xL [2]. In murine cardiac fibroblasts, miRNA (miR)-21 modulates matrix metalloproteinase-2 expression at the infarct zone via a phosphatase and tensin homologue pathway [3]. However, the dynamic features in gene expressions, biological functions and regulatory interactions in MI progression have not been fully studied at a system level.

Dynamic data could provide more valuable information for understanding biological processes than data focused on single time points [4–6]. Nowadays, different genomics data can be measured over time, of which time-series gene expression data are one of the most abundant and available data [4]. Recently, some studies have applied time-series gene expression data to uncover complex regulatory mechanisms underlying biological process and disease status [6–11]. For example, Nazarov *et al*. [7, 8] systematically investigated dynamic regulation of mRNA and miRNA expression following stimulation of melanoma cells with interferon-γ, and revealed dynamic interplay of miRNA and upstream regulators with biological functions in human. Li *et al*. [10] identified dynamic network biomarkers and analyzed the underlying mechanisms of complex diseases by constructing dynamic human protein–protein interaction networks. For MI, Port *et al*. [6] analysed changes in expression of mRNAs and miRNAs in mouse using expression profiles of three time points post-MI. Zhang *et al*. [11] identified several key genes and their possible functions in acute MI by the analysis of six time points gene expression data in mouse. However, the dynamic characteristics of biological function and regulatory interactions in MI progression in the above two studies have not been investigated, and whether the results in mouse are applicable to humans remains unknown.

Network-based systems biology approaches have emerged as powerful tools for deciphering the complex regulatory interactions underlying diseases [12, 13]. Gene regulatory networks control gene expression and protein formation, and therefore influence the cell fate. miRNAs and transcription factors (TFs) are two major regulators in gene regulatory networks and are involved in many important biological events, such as cell proliferation, differentiation and apoptosis. Therefore, abnormal expression of miRNAs and TFs will trigger a series of diseases. MiRNAs are small non-coding RNAs (~22nt) capable of inhibiting expression of target mRNAs by binding to their 3' untranslated regions (UTRs) [14]. MiRNAs regulate expression of genes at the post-transcriptional level, while TFs modulate gene expression at the transcriptional level. Additionally, miRNAs and TFs can regulate the same target genes, and they may mutually regulate each other; hence forming feed-forward loops (FFLs), which have been demonstrated to comprise recurrent network motifs and play important roles in mammalian genomes [15, 16]. Recently, Zhang *et al*. [17] and Arora *et al*. [18] have reviewed miRNA–TF co-regulatory loops in biological processes and diseases. The miRNA and TF interactions focused on FFLs in cancer have been studied [19–23]. For example, significant miRNA–TF FFLs associated with cancer have been identified using mRNA and miRNA expression profiles [21]. The gene regulatory networks involved in miRNA–TF FFLs have been examined for breast cancer subtypes to study their distinct and common features [22]. Simultaneously, MI-related miRNA–TF co-regulatory network based on FFLs has also been investigated [24, 25], from which certain important regulators and regulatory modules were identified. However, these studies were focused on static networks, but the regulatory interactions within the networks vary with different disease states. Thus, dynamic miRNA–TF co-regulatory networks involved in FFLs are essential to capture the changing information during disease progression.

**Fig 1:**
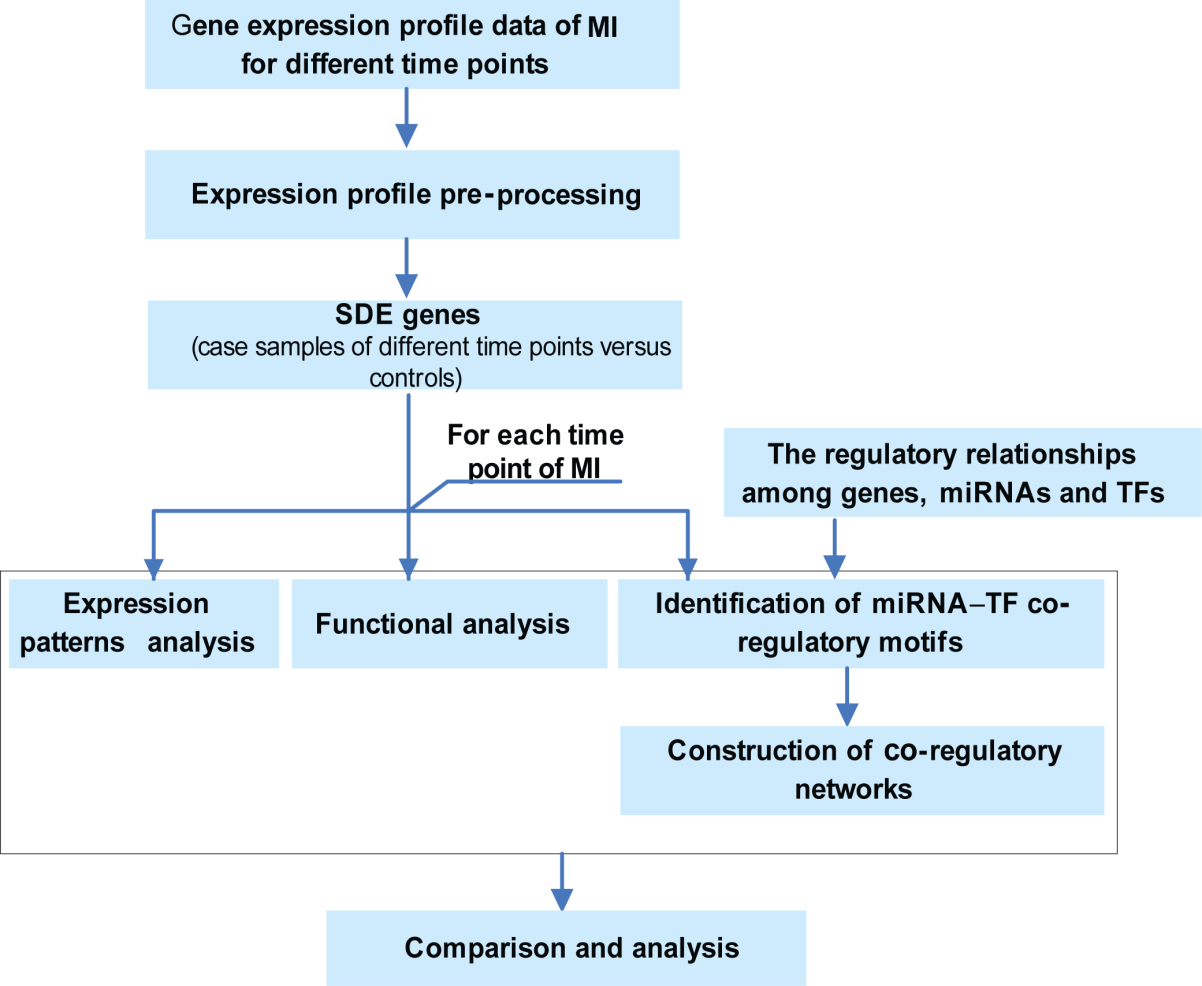
The schematic representation of the computational analysis pipeline.

In this study, we revealed the dynamic features in MI progression at a system level via integrating time-series gene expression data and miRNA–TF co-regulatory networks analysis. The workflow was depicted in Fig 1. Three aspects were investigated including dynamic changes in expression, biological function and the regulatory interactions among miRNAs, TFs and target genes. Additionally, a new panel of diagnostic biomarkers defined by STAT3 and ICAM1 was identified.

## Materials and Methods

### Gene Expression Profiles

The MI related gene expression profile data were downloaded from the Gene Expression Omnibus (GEO) database under accession number of GSE62646 (www.ncbi.nlm.nih.gov/geo/query/acc.cgi?acc=GSE62646) [26], including a case group of 28 patients with ST-segment elevation MI and a control group of 14 patients with stable coronary artery disease, without a history of MI. The gene expression profiles were measured in peripheral blood mononuclear cells drawn from all samples, and the blood was collected on day 1 of MI (acute phase), after 4–6 days (subacute phase), and after 6 months (chronic phase). More detailed information on samples collection and diagnosis criteria can be obtained from reference [26].

### Expression Profile Pre-Processing and Screening of Differentially Expressed Genes

To reduce unreliable information, primary data of microarray expression profiles were pre-processed according to previous studies [7, 8]. Probes were removed for which the maximum expression over all arrays did not reach a signal intensity of 7 on a log_2_ scale. The probes were removed when one probe corresponded to multiple genes. Expression values were averaged when multiple probes corresponded to one gene [27–29]. We then retained protein-coding genes. To identify significantly differentially expressed (SDE) genes comparing expression data in case samples with controls, differential analysis was performed using the R ‘limma’ package. To control the false discovery rates, the Benjamini–Hochberg multiple testing correction was conducted. The genes with adjusted *p*<0.001 and >1.2-fold change were selected as SDE genes.

### Enrichment Analysis

Enrichment analysis of SDE genes (case samples versus controls) at each time point was implemented in the following two steps: (1) KEGG pathway enrichment analysis for SDE genes was performed using DAVID; and (2) KEGG subpathway (local area of the entire biological pathway) enrichment analysis for SDE genes was implemented using the R ‘SubpathwayMiner’ package [30]. Significantly enriched pathways and subpathways were identified with a Benjamini–Hochberg adjusted *p*<0.05 (S1 Fig and S1 File). In order to show the results more clearly, if multiple significantly enriched subpathways corresponded to an entire pathway, the subpathway with the minimum adjusted *p* value was retained.

### Identification of miRNA–TF Co-Regulatory Motifs

MiRNA–TF co-regulatory motifs are known to play important roles in gene regulation [15, 16]. A miRNA–TF co-regulatory motif is one in which a TF and a miRNA regulate each other and they both regulate a common target gene (S2 Fig). Starting from SDE genes (case samples versus controls), experimentally verified regulatory relationships among miRNAs, TFs and target genes were identified as follows. First, miRNA-gene regulatory relationships supported by strong experimental evidence (e.g. western blotting, reporter assay and qRT-PCR) were extracted from TarBase (version 6.0) [31], miRTarBase (version 4.5) [32] and miRecords (version 4) [33] databases. MiRNAs were mapped to mature miRNAs based on miRBase (release 21) [34]. Second, to obtain regulatory relationships of miRNA–TF, a list of 1698 unique human TFs was retrieved from a previous study [25] and the above procedure was implemented, thus the relationships between miRNAs and TFs were determined. Third, experimentally confirmed TF-gene regulatory relationships were collected from TRED [35] and Transfac (April 2012) [36] databases, and experimentally confirmed TF-miRNA regulatory relations were retrieved from TransmiR (version 1.2) [37].

### miRNA–TF Co-Regulatory Networks Construction and Analysis

MiRNA-TF co-regulatory networks were constructed by combining identified miRNA–TF co-regulatory motifs. For each time point of MI, the miRNA-TF co-regulatory networks were then obtained. To evaluate the importance of a node in the co-regulatory network, degree and betweenness centrality were used as measurements. Degree of a node was the number of edges connected to it. A node was defined as a hub using the method proposed by Yu et al [38]. For a node *i* in the network, the betweenness centrality (BC) was defined as $BC_{i}=\sum_{s\neq i\neq t}\frac{\sigma_{i}(s,t)}{\sigma (s,t)}$, where *σ*(*s*, *t*) was the total number of shortest paths from node *s* to node *t*, and *σ*_*i*_(*s*, *t*) was the number of those paths that pass through node *i*. The R VennDiagram package was used to plot the Venn diagrams for comparing miRNA–TF co-regulatory networks, and the common and specific nodes and edges in the co-regulatory networks were then analyzed.

### Classification of Diagnostic Biomarkers

To examine the classification efficiency of the biomarkers in distinguishing controls from patients, and patients with recurrent events from those without recurrent events, a classification model based on Naive Bayes was implemented using the Weka system. Naive Bayes classifier [39] is one of the most commonly used classifier which is a probabilistic statistical classifier based on Bayes' theorem, and it has been successfully used for clustering and classification in biomedical research [40, 41]. The area under the receiving operating curve (AUC) was used to evaluate performance according to previous studies [41, 42]. An AUC value ranges from 0 to 1, with 0.5 indicating random performance and 1.0 implying perfect predictive performance. The gene expression profile data of GSE62646 mentioned above were used as training set. The gene expression profile data of GSE48060 from the GEO database were used as an independent test set, which included 21 normal cardiac function controls and 31 acute MI patients. Among the 31 patients, 27 had follow-up data, including five with recurrent events and 22 without any recurrent events over 18-months follow-up. Blood samples from the 21 controls and 31 patients within 48-h post-MI were used for producing gene expression profiles.

## Results

### Differentially Expressed Genes

After pre-processing of the expression profiles, 8741 protein-coding genes remained for further analysis. We then identified SDE genes (case samples of three time points versus controls) using the R ‘limma’ package, resulting in 1970 SDE genes over all time points. A total of 1611, 1098 and 939 SDE genes for acute phase, subacute phase and chronic phase of MI were obtained separately (S2 File), and 618 SDE genes were shared by them.

### Dynamic Expression Patterns of SDE Genes

**Fig 2:**
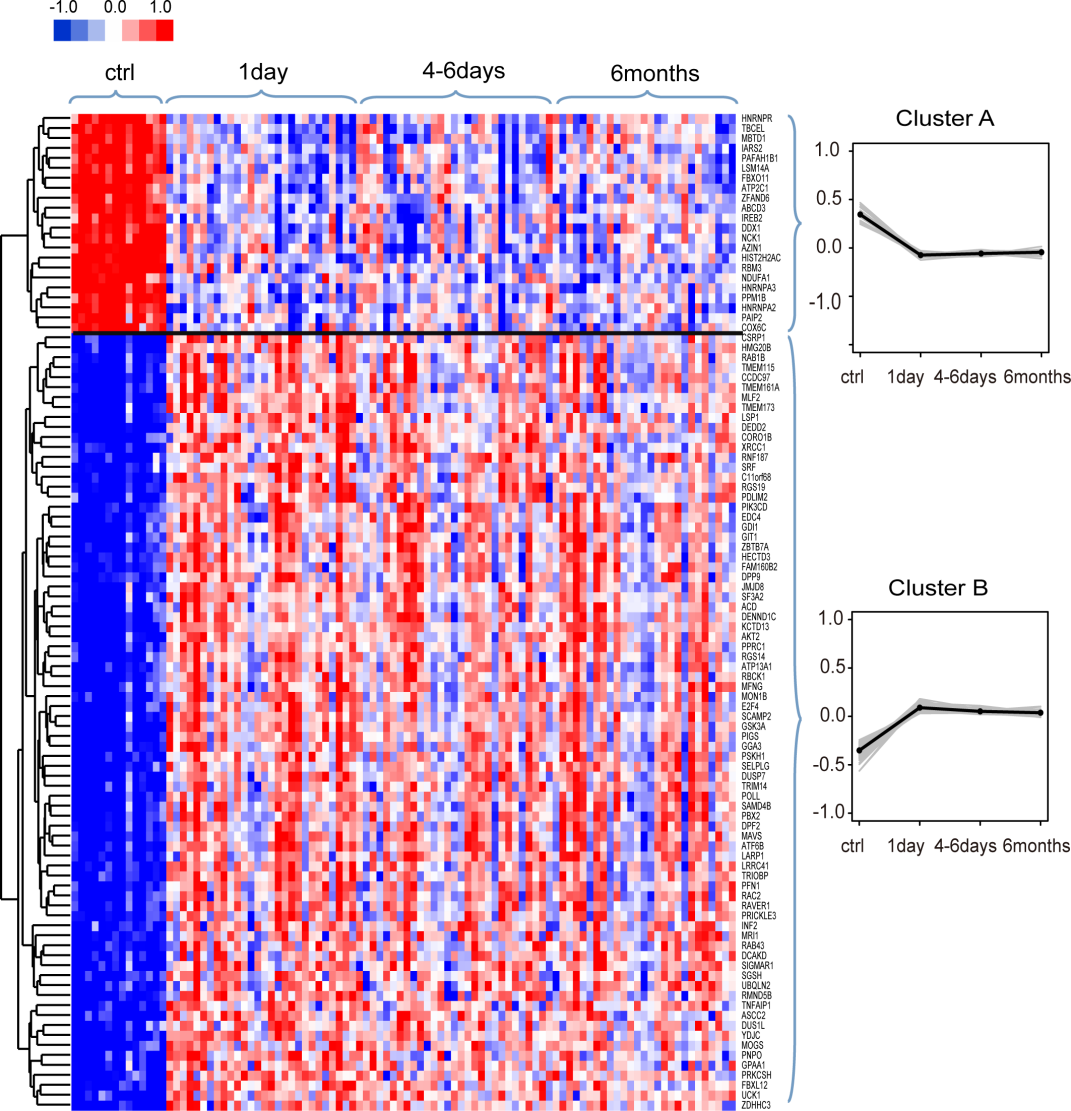
Hierarchical clustering on standardized expression values of the top 100 SDE genes. Two main clusters were identified, and each cluster included its corresponding gene profiles (grey lines) and mean expression values (dark dots).

We investigated the dynamic changes in expression of the SDE genes at three time points. First, the overall expression changes of these SDE genes were investigated. To demonstrate the results more clearly, we only show the top 100 SDE genes. Further studies were implemented using all SDE genes. Hierarchical clustering on standardized expression values using Cluster3.0 software [43, 44] by the city-block distance and complete linkage method was performed. As shown in Fig 2 (shown by JavaTreeView), two main clusters were identified. Cluster A displayed rapid down-regulation reaching its lowest point on day 1 after MI, and then slightly increased until 6 months. In contrast, cluster B demonstrated marked up-regulation reaching a plateau on day 1 after MI, and then decreased slowly until 6 months.

Next, dynamic changes in expression of individual SDE genes were analyzed. We focused on the top 1% SDE genes (six genes), and expression changes of these six genes (MLF2, KCTD13, AKT2, GSK3A, DEDD2 and YDJC) were shown in S3 Fig. Although these six genes all showed a rapid up-regulation after MI, the trend in changes in expression during subsequent time points was different. For example, MLF2 remained down-regulation until 6 months, while KCTD13 displayed decreased expression until 4–6 days and then increased expression until 6 months.

### Dynamically Functional Analysis of SDE Genes

To investigate dynamic functional changes affected by SDE genes over time, significantly enriched KEGG biological pathways were identified using DAVID [45] based on SDE genes (case samples of three time points compared with controls) (S3 Fig). To compare the functional changes at different times, colouring was implemented according to adjusted *p* values. For each pathway, the smallest adjusted *p* value was coloured black, and white denoted that the adjusted *p* value was not significant or the SDE genes were not enriched in this pathway. Although some well-known and important pathways in MI were identified, including the TGF-β signalling pathway and apoptosis, the significantly enriched biological pathways were few.

**Fig 3:**
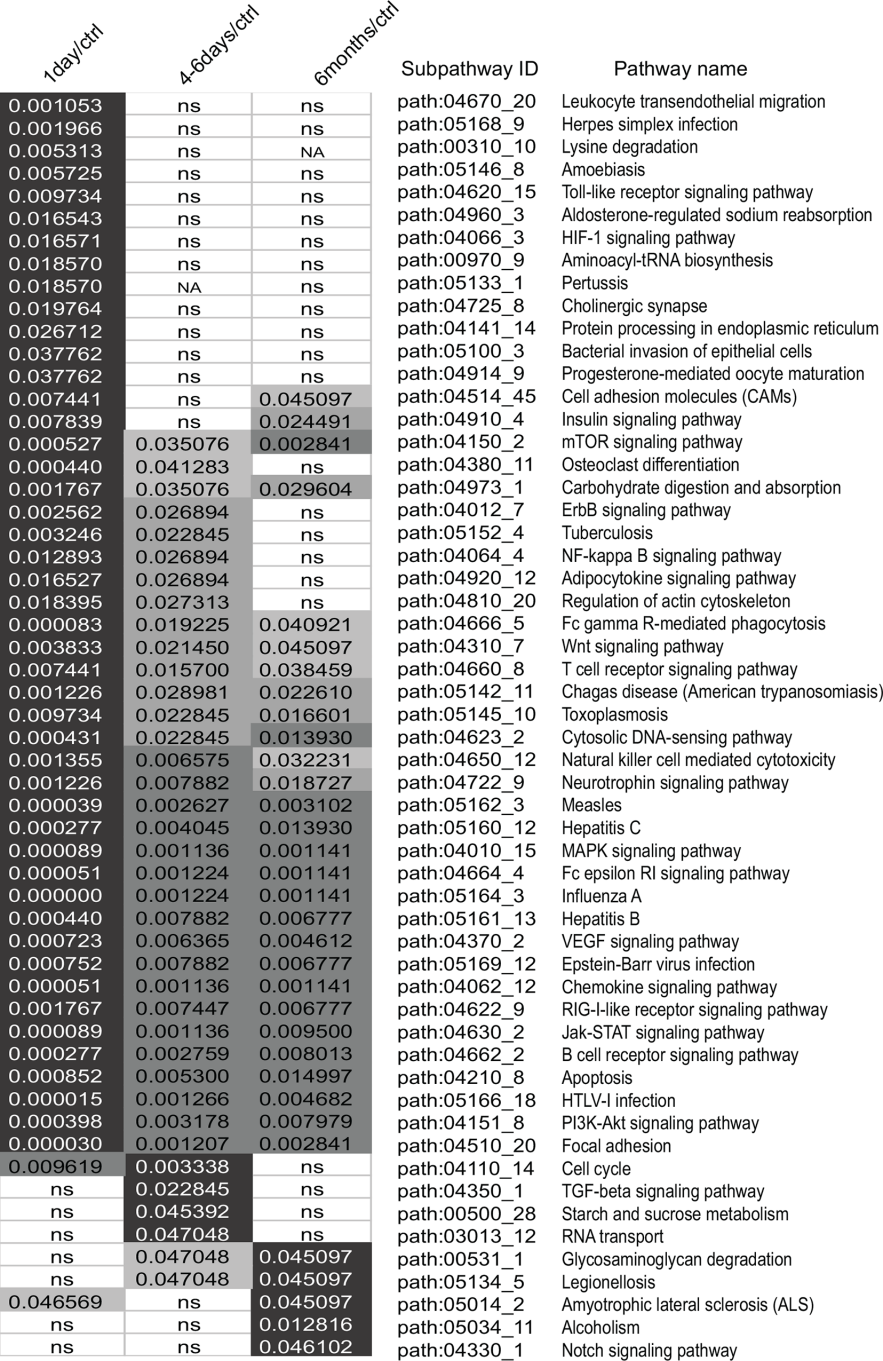
Dynamic changes of subpathways based on SDE genes. Significantly enriched subpathways (cancer subpathways removed) were obtained using SubpathwayMiner. Colouring was performed based on adjusted *p* values: black–the smallest adjusted *p* value; white–non-significant (ns, >0.05) or SDE genes were not enriched in this pathway (NA).

Previous studies have demonstrated that some type-specific functions tend to be distributed in local areas of the pathway (subpathway) instead of the entire pathway, and thus subpathway may provide more detailed explanations for pathogenesis [30, 46]. We therefore used SubpathwayMiner [30] to identify significantly enriched KEGG subpathways, and 69 significant subpathways were reported (S4 Fig). To illustrate the results more clearly, cancer pathways were removed. As shown in Fig 3, the functional pathways were classified into three main categories. Each category had pathways in common with the others, and each category had its own specific pathway. The first and the largest group contained early cellular reactions at the onset of MI, including leukocyte transendothelial migration, the Toll-like receptor signaling pathway, the NFκB signaling pathway, the chemokine signaling pathway and apoptosis, suggesting inflammation, immune response and cell apoptosis. The second group displayed cellular responses in the subacute phase, including the TGF-β signalling pathway, which are known to play crucial roles in cardiac repair and remodeling [47, 48]. Finally, several pathways such as alcoholism, glycosaminoglycan degradation, legionellosis, amyotrophic lateral sclerosis and the Notch signaling pathway were enriched in the chronic phase of MI. Additionally, we noted that certain pathways were shared by these three groups, such as the chemokine signaling pathway and the VEGF signaling pathway.

### Dynamic Features of Regulatory Interactions among miRNAs, TFs and Target Genes in MI Progression

**Fig 4:**
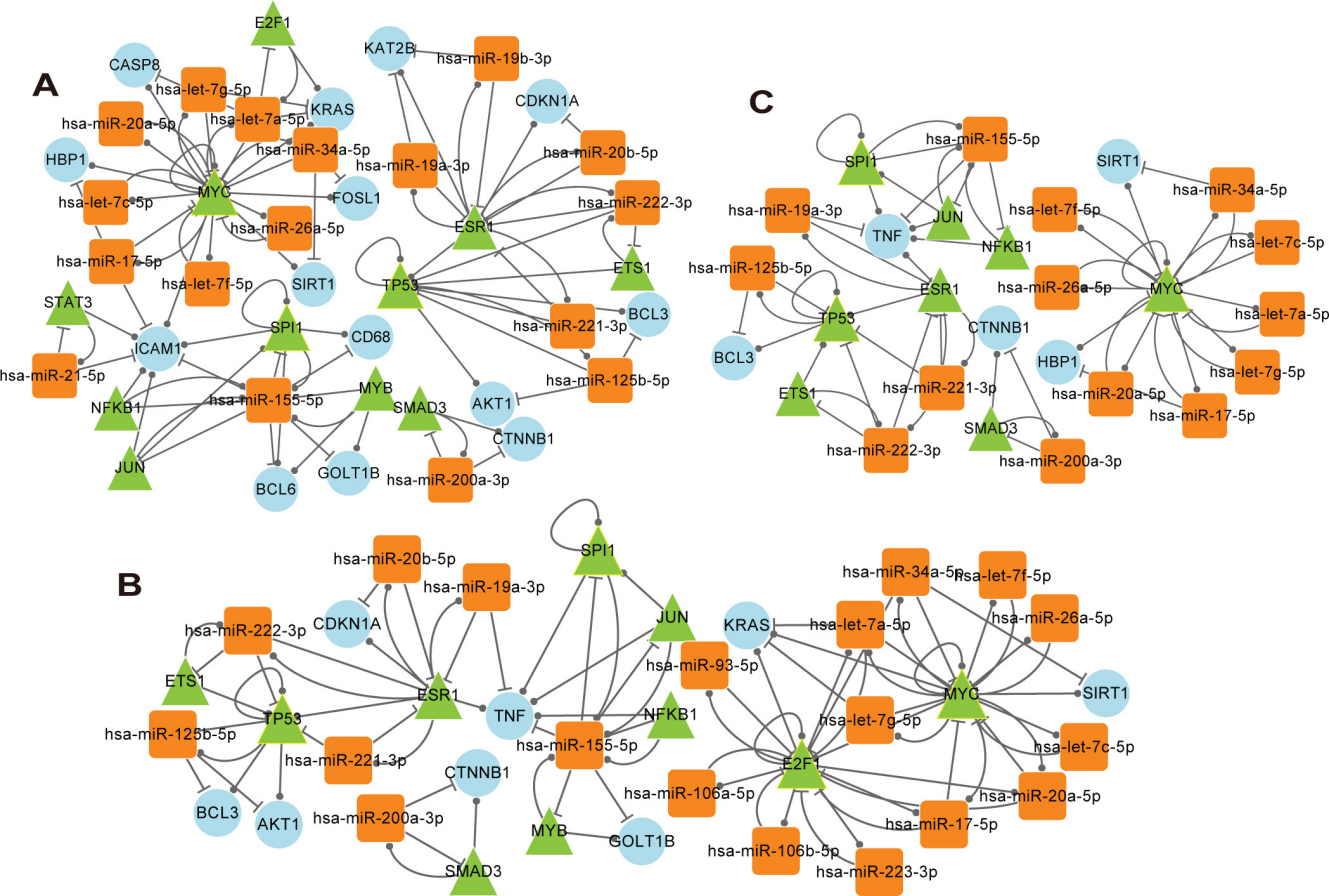
MiRNA–TF co-regulatory networks in MI progression. (A). Acute phase. This network consisted of 42 nodes and 90 links. (B). Subacure phase. This network consisted of 37 nodes and 85 links. (C). Chronic phase. This network consisted of 27 nodes and 56 links. Round rectangles denote miRNAs, circles denote genes, and triangles denote TFs. The nodes with yellow border indicate both genes and TFs.

To study the dynamic features of regulatory interactions among miRNAs, TFs and target genes, dynamic miRNA–TF co-regulatory networks for MI were constructed. For each time point of MI, miRNA–TF co-regulatory motifs were identified (Table 1) and then they were merged to construct the miRNA–TF co-regulatory networks (see Materials and methods). As demonstrated in Fig 4, the network of the acute phase was the largest, and it contained 42 nodes (17 miRNAs, 11 TFs and 17 genes) and 90 edges. Among 11 TF and 17 genes, three common nodes (MYC, TP53 and SPI1) were observed. The network of the subacute phase contained 37 nodes (19 miRNAs, 10 TFs and 12 genes) and 85 edges. Among 37 nodes, four nodes (MYC, TP53, SPI1 and E2F1) were common to TFs and genes. The network of the chronic phase only contained 27 nodes (14 miRNAs, eight TFs and eight genes) and 56 edges. Among 27 nodes, three nodes (MYC, TP53 and SPI1) were shared by TFs and genes.

**Table 1:** Summary of miRNA–TF co-regulatory motifs at three time points of MI.

		Number of nodes				Number of links				
Time point	Number of motifs	miRNAs	TFs	Genes	Total	miRNA-gene	miRNA-TF	TF-gene	TF-miRNA	Total
1 day	36	17	11	17	42	30	22	26	22	90
4–6 days	38	19	10	12	37	30	26	20	26	85
6 months	22	14	8	8	27	18	17	14	17	56

For each miRNA–TF co-regulatory network, degree and betweenness centrality were calculated to evaluate the importance of a node in maintaining the overall connectivity of the network (S3 File). The nodes with higher degree tended to have higher betweenness centrality. Although the three networks shared the hub miRNA (hsa-miR-155-5p and hsa-miR-222-3p) and hub TF (MYC, ESR1, TP53 and SPI1), the genes regulated by them and the genes regulating them were different (Fig 4). For example, the degree of hsa-miR-155-5p and MYC in the three networks was decreased. Taking hsa-miR-155-5p as an example, its degree was 12, 10 and 7 in the three networks, respectively. Three genes (BCL6, CD68 and ICAM1) were regulated by hsa-miR-155-5p in the acute phase but not subacute and chronic phases, whereas TNF was regulated by hsa-miR-155-5p in the subacute and chronic but not acute phase. Four TFs (JUN, MYB, NFκB1 and SPI1) regulated hsa-miR-155-5p in all the three phases, except that MYB was absent in the chronic phase. These results indicated that, although the different stages after MI shared common hub miRNAs and hub TFs in their miRNA–TF co-regulatory networks, they possessed different regulatory relationships via connection with different genes, which might lead to the different biological processes and phenotypic characteristics.

**Fig 5:**
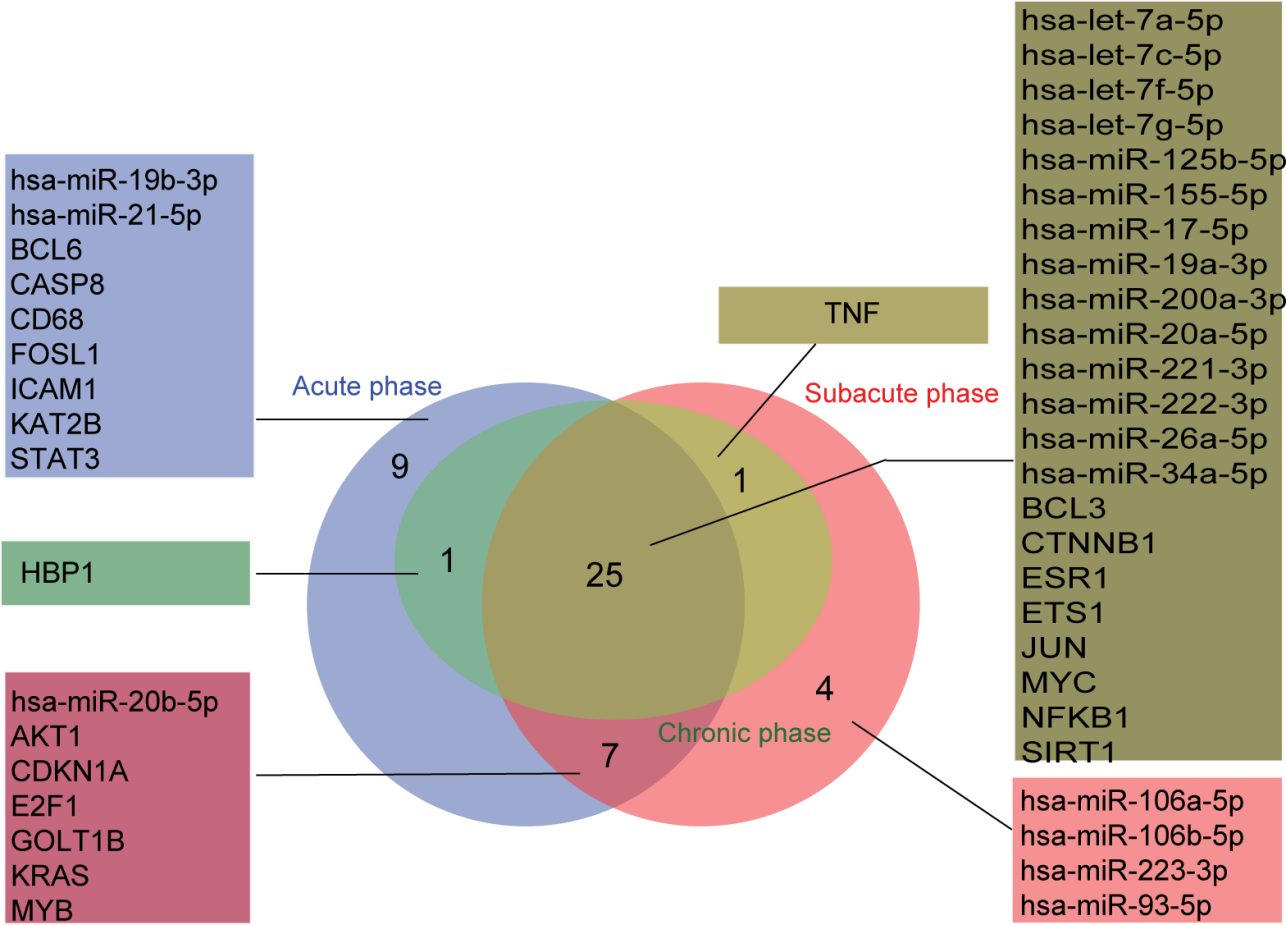
Comparison of nodes in miRNA–TF co-regulatory networks in MI progression (acute, subacute and chronic phases).

**Fig 6:**
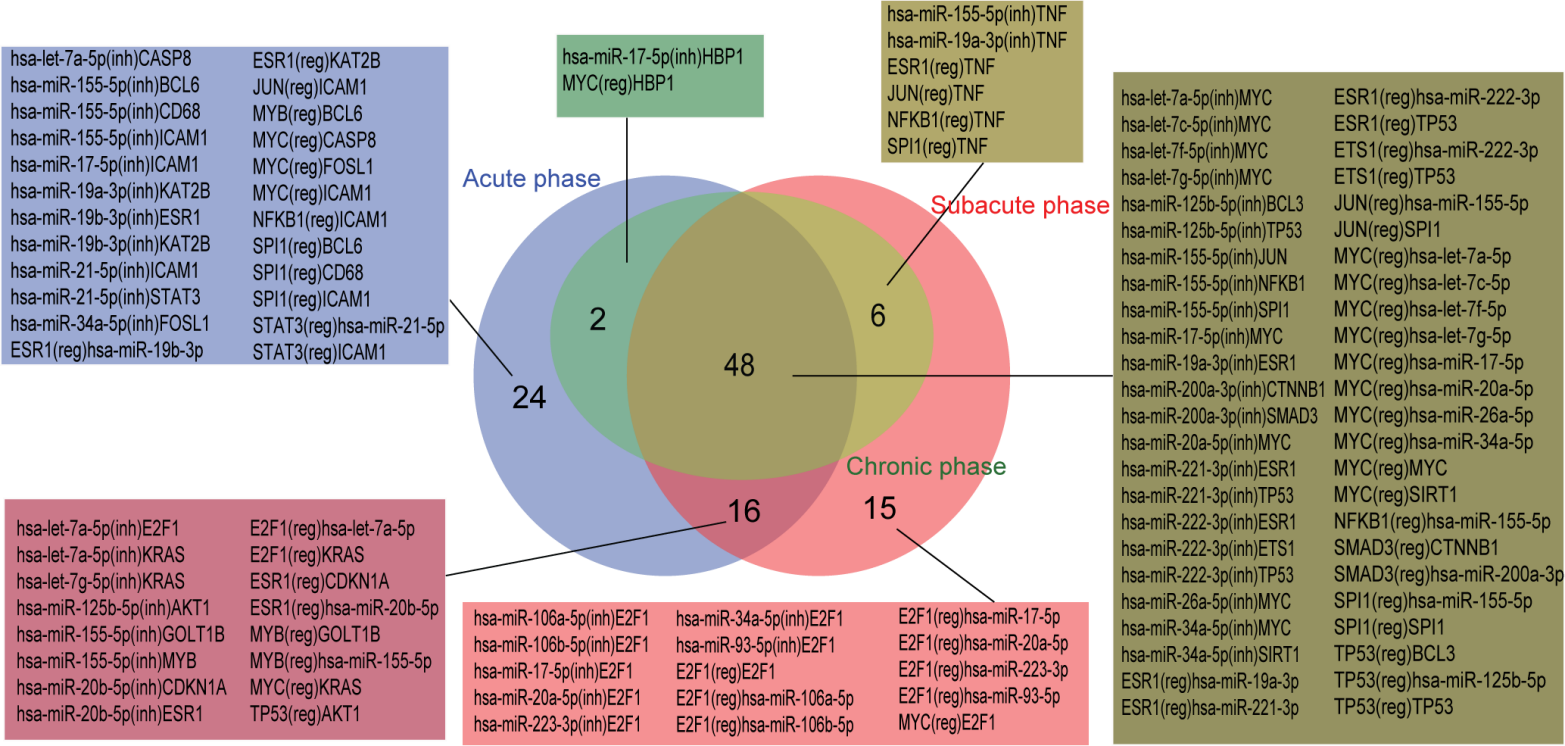
Comparison of edges in miRNA–TF co-regulatory networks in MI progression (acute, subacute and chronic phases). ‘inh’ indicated inhibition, ‘reg’ indicated regulation.

The nodes and the edges in each miRNA–TF co-regulatory network were compared, and common and unique nodes and edges were identified using the R VennDiagram package. As shown in Figs 5 and 6, the network of the chronic phase was contained in the network of the acute and subacute phases. For nodes, the three stages together enjoyed 25 nodes including hsa-miR-155-5p, MYC and NFκB1. Nine nodes were found only in the acute phase, and four nodes were found only in the subacute phase. For edges, 48 common edges were found in the three stages. 24 edges were specific for the acute phase and 15 for the subacute phase.

**Fig 7:**
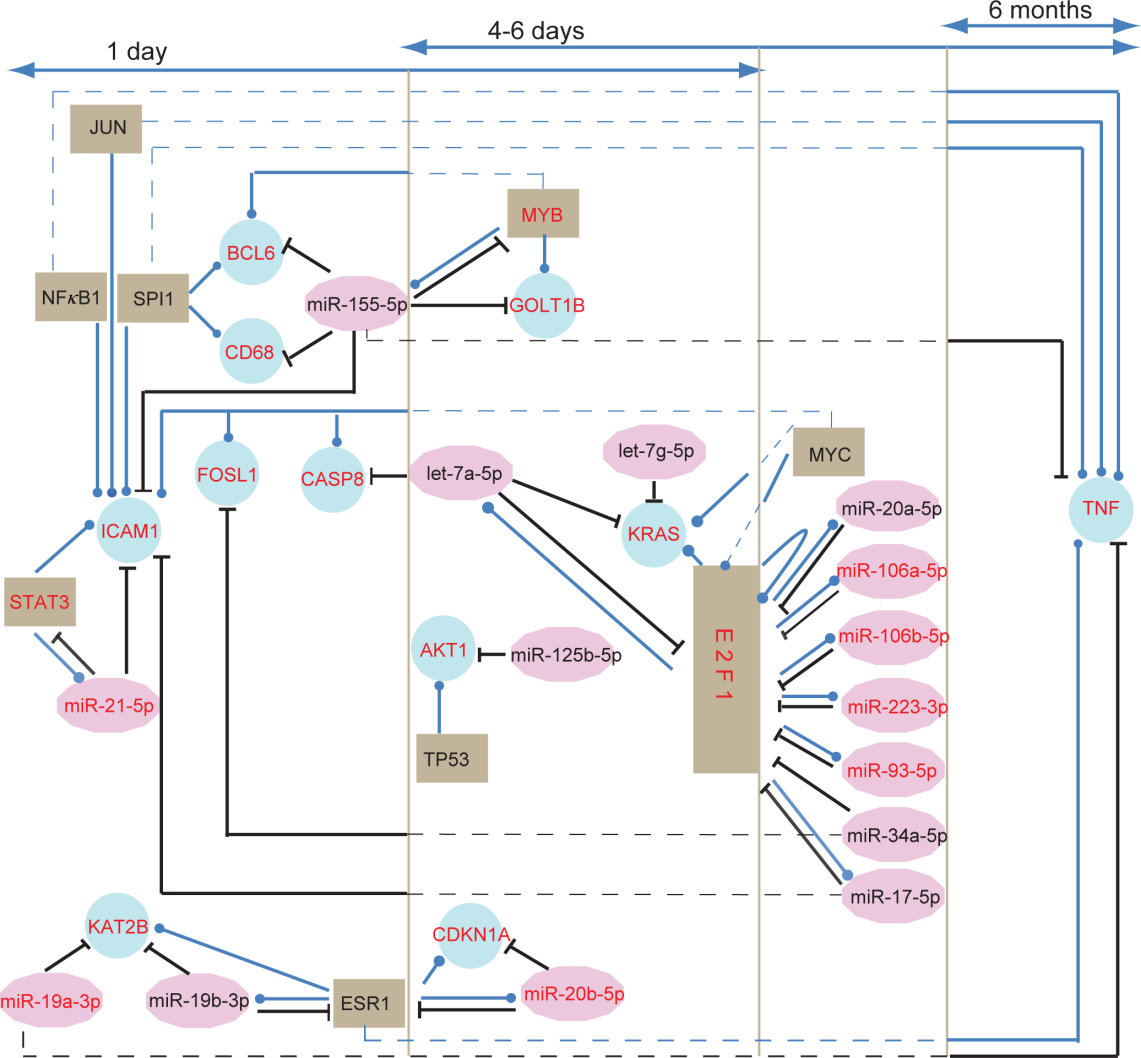
Graphical representation of dynamic regulatory interactions among miRNAs, TFs and target genes in MI progression based on stage-specific nodes and edges. The nodes with red symbols represented the stage-specific nodes. For example, KAT2B was the unique gene in the acute phase, and MYB was the specific gene in the acute and subacute phases. The edges represented by solid lines were stage-specific, while the edges represented by dotted lines were not found in corresponding stages. For example, E2F1 regulation miR-106b-5p was specific in the subacute phase, and TNF was inhibited by miR-155-5p only in the subacute and chronic phases.

Furthermore, we examined the stage-specific genetic factors implicated with the development of MI in more detail. Regulatory interactions among miRNAs, TFs and target genes in three phases of MI were then extracted based on the specific nodes and edges (Fig 7), and regulatory interactions among miRNAs, TFs and target genes were analyzed. In the acute phase, ICAM1 which was a specific gene, was specifically controlled by multiple miRNAs (miR-21-5p, miR-17-5p and miR-155-5p) and TFs (STAT3, NFκB1, JUN, SPI1 and MYC). Meanwhile, miR-21-5p, STAT3 and ICAM1 formed a network motif in which the nodes and the edges were all specific. In addition, the unique node KAT2B was specifically regulated by miR-19a-3p and miR-19b-3p. At transition stage from acute to subacute phase, three motifs (miR-155-5p, MYB, GOLT1B; let-7a-5p, E2F1, KRAS and miR-20b-5p, ESR1, CDKN1A) were found, in which all the edges and at least two nodes were specific. In the subacute phase, E2F1 not only specifically regulated itself, but also modulated four specific miRNAs (miR-106a-5p, miR-106b-5p, miR-223-3p and miR-93-5p) each other. At the transition from the subacute to chronic phase, TNF was the only unique gene that was specifically regulated by two miRNAs (miR-155-5p and miR-19a-3p) and four TFs (NFκB1, JUN, SPI1 and ESR1). This suggested that certain regulators controlled the expression of certain genes during different stages of MI progression, and this triggered the different pathological mechanisms and clinical features.

### Identification of Candidate Diagnostic Biomarkers in MI

Based on the above findings, we found that the motif composed of miR-21-5p, STAT3 and ICAM1 was both specific for nodes and edges, which might play important roles in MI diagnosis. Thus, the classification model was implemented (see Materials and methods). Since the matched miRNA expression profiles were not available, we used the signature defined by STAT3 and ICAM1 as inputs to the Naive Bayes model, and an AUC value of 0.880 was obtained in the training set by applying leave-one-out cross validation analysis. The signature was also tested in an independent test set (GSE48060), and the classification performance was satisfactory (AUC = 0.713). In addition, we investigated the potential ability of STAT3 and ICAM1 in predicting recurrence of MI by applying expression profiles of GSE48060. As a result, an AUC value of 0.795 was obtained (Table 2).

**Table 2:** Classification performance of the combination of STAT3, ICAM1 and single STAT3 or ICAM1 based on leave-one-out cross validation analysis.

Inputs	STAT3, ICAM1	STAT3	ICAM1
*Training set (AUC)	0.880	0.821	0.842
*Independent test set (AUC)	0.713	0.718	0.741
^△^**Independent test set (AUC)**	0.795	0.765	0.678

Note: ‘*’ denotes the data set for distinguishing patients with or without MI. ‘^△^’ indicates the data set for distinguishing MI patients with or without recurrent events.

## Discussion

Dynamic data of a biological system could provide more valuable information in elucidating complex gene regulation mechanisms underlying biological processes than data of single conditions. In this study, we systematically investigated the dynamic features in MI progression by integrating miRNA–TF co-regulatory networks and time-series gene expression data. Dynamic expression patterns were analyzed and biological functions associated with each MI stage were identified. Additionally, dynamic interplay of miRNAs, TFs and target genes were analyzed, and a new candidate diagnostic signature defined by STAT3 and ICAM1 was identified.

We identified different biological pathways at different MI stage. For chronic phase of MI, several pathways such as alcoholism, glycosaminoglycan degradation, legionellosis, amyotrophic lateral sclerosis and the Notch signaling pathway were enriched. Among these pathways, the alcoholism pathway was significantly enriched, and acute MI triggered by alcohol consumption has been reported [49]. So it is possible that alcoholism is related with heart disease, and heart disease patients may be more inclined to make dietary changes to prevent occurrence of future infarcts. The adjusted *p* values of the other four pathways (glycosaminoglycan degradation, legionellosis, amyotrophic lateral sclerosis and Notch signaling pathway) were marginally significant. However, previous studies have shown that glycosaminoglycans are key molecules in atherosclerosis [50] and Notch signalling is an important mediator of cardiac repair and regeneration after MI [51]. Legionellosis and amyotrophic lateral sclerosis have complex relationships with MI [52, 53].

The dynamic miRNA–TF co-regulatory networks were constructed based on experimentally verified regulatory relationships among miRNAs, TFs and target genes. Here, we focused on the accuracy but not the coverage, and thus the predicted data were not used. However, the experimentally confirmed regulatory relationships were neither complete nor unbiased, and these data did not include time point information that might affect the accuracy of the networks. With an improvement of the quantity and quality of these data and the availability of matched mRNA, miRNA and long non-coding RNA expression profiles measured at multiple time points, the dynamic features in MI progression will be comprehensively and accurately researched at a system level.

The classification performances of single STAT3 or ICAM1 were also tested. For distinguishing patients with or without MI, both single STAT3 and ICAM1 obtained lower AUC values in the training set (0.821 for STAT3 and 0.842 for ICAM1) and a bit higher AUC values in the testing set (0.718 for STAT3 and 0.741 for ICAM1) than their combinations (Table 2). For distinguishing MI patients with or without recurrent events, both single STAT3 and ICAM1 obtained lower AUC values (0.765 for STAT3 and 0.678 for ICAM1) than their combinations (Table 2). Simultaneously, we noted that the dataset available for performance evaluation was limited, and confirmation in a large independent cohort and experimental verification are warranted. Additionally, gene expression profile data we used was measured from peripheral blood mononuclear cells but not from myocardial tissues. This means that the dynamic features we obtained may be indirectly caused by MI.

Our research revealed the dynamic architecture and features in MI progression at several levels, including expression, biological function, and regulatory interactions at the transcriptional and post-transcriptional levels. Especially, a new panel of candidate diagnostic biomarkers defined by STAT3 and ICAM1 was identified to have discriminatory capability for patients with or without MI, especially the patients with or without recurrent events. All these results provide important clues for greater understanding of the dynamic regulatory mechanisms in MI progression, and could provide new diagnostic biomarkers for MI.

## Supporting Information

S1 Fig Dynamic changes of pathways based on SDE genes. Significantly enriched pathways were obtained using DAVID. Colouring was performed based on adjusted p values: black–the smallest adjusted *p* value; white–non-significant (ns, > = 0.05) or SDE genes were not enriched in this pathway (NA).
(TIF)

S2 Fig A miRNA–TF co-regulatory motif. A TF and a miRNA regulate each other and they both regulate a common target gene.
(TIF)

S3 Fig Expression changes of top 1% SDE genes (six genes). The y-axis represented standardized expression values, while the x-axis represented time points.
(TIF)

S4 Fig Dynamic changes of subpathway based on SDE genes. Significantly enriched subpathways were obtained using SubpathwayMiner. Colouring was as described above.
(EPS)

S1 File Significantly enriched subpathways. (XLS)

S2 File Differentially expressed genes. (XLSX)

S3 File Degree and betweenness centrality of each node in the miRNA–TF co-regulatory networks. (XLSX)
