# Supplementary figures and images for: Studying Dynamic Features in Myocardial Infarction Progression by Integrating miRNA-Transcription Factor Co-Regulatory Networks and Time-Series RNA Expression Data from Peripheral Blood Mononuclear Cells
Source: PLoS One. 2016 Jul 1;11(7):e0158638. doi: 10.1371/journal.pone.0158638 (PMC4930172; doi:10.1371/journal.pone.0158638)

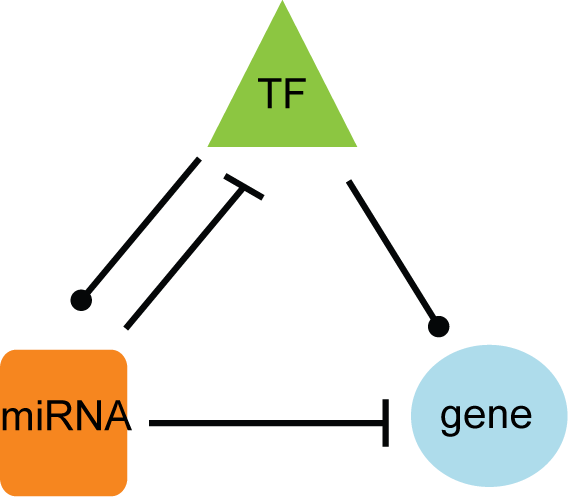

Supplement: S1 Fig — Significantly enriched pathways were obtained using DAVID. Colouring was performed based on adjusted p values: black–the smallest adjusted p value; white–non-significant (ns, > = 0.05) or SDE genes were not enriched in this pathway (NA). (TIF) [file pone.0158638.s001.tif]

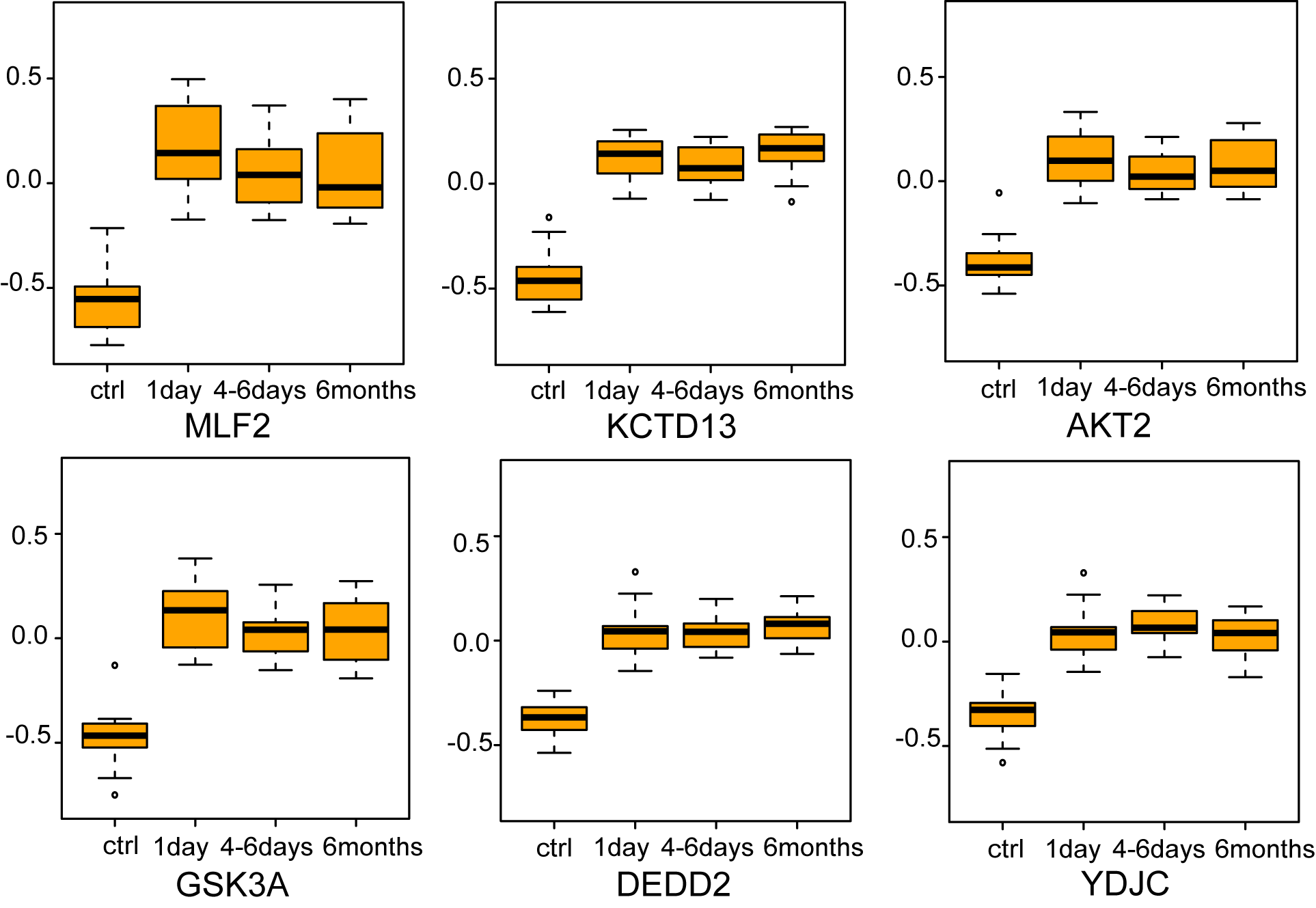

Supplement: S2 Fig — A TF and a miRNA regulate each other and they both regulate a common target gene. (TIF) [file pone.0158638.s002.tif]

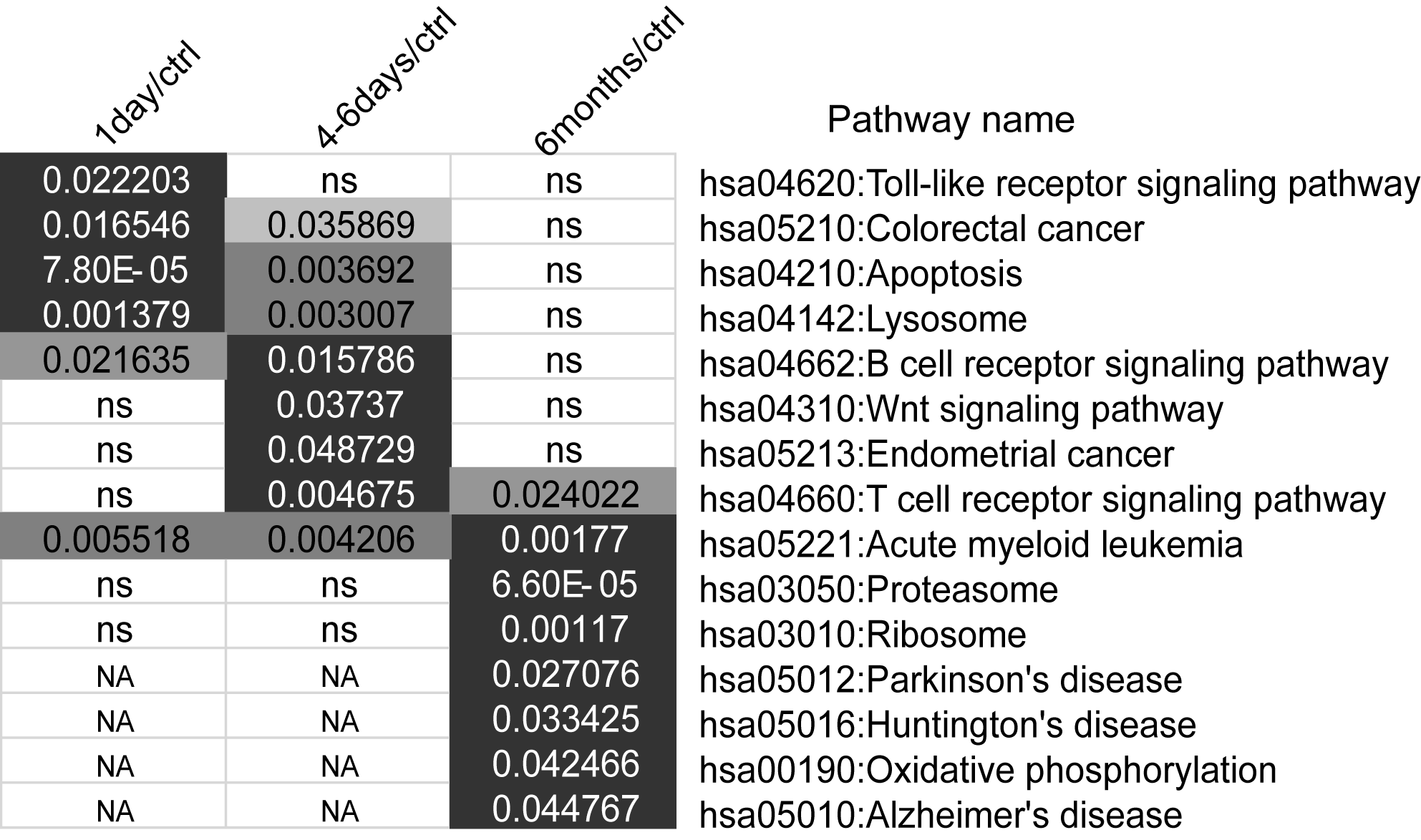

Supplement: S3 Fig — The y-axis represented standardized expression values, while the x-axis represented time points. (TIF) [file pone.0158638.s003.tif]
